# Supplementary material for: Oxidative Stress and Its Modulation by Ladostigil Alter the Expression of Abundant Long Non-Coding RNAs in SH-SY5Y Cells
Source: Noncoding RNA. 2022 Oct 25;8(6):72. doi: 10.3390/ncrna8060072 (PMC9680243; doi:10.3390/ncrna8060072)
Supplement: Supplementary file 1 [file ncrna-08-00072-s001.zip › Supplementary Figure S1.pdf]

Supplementary Figure S1

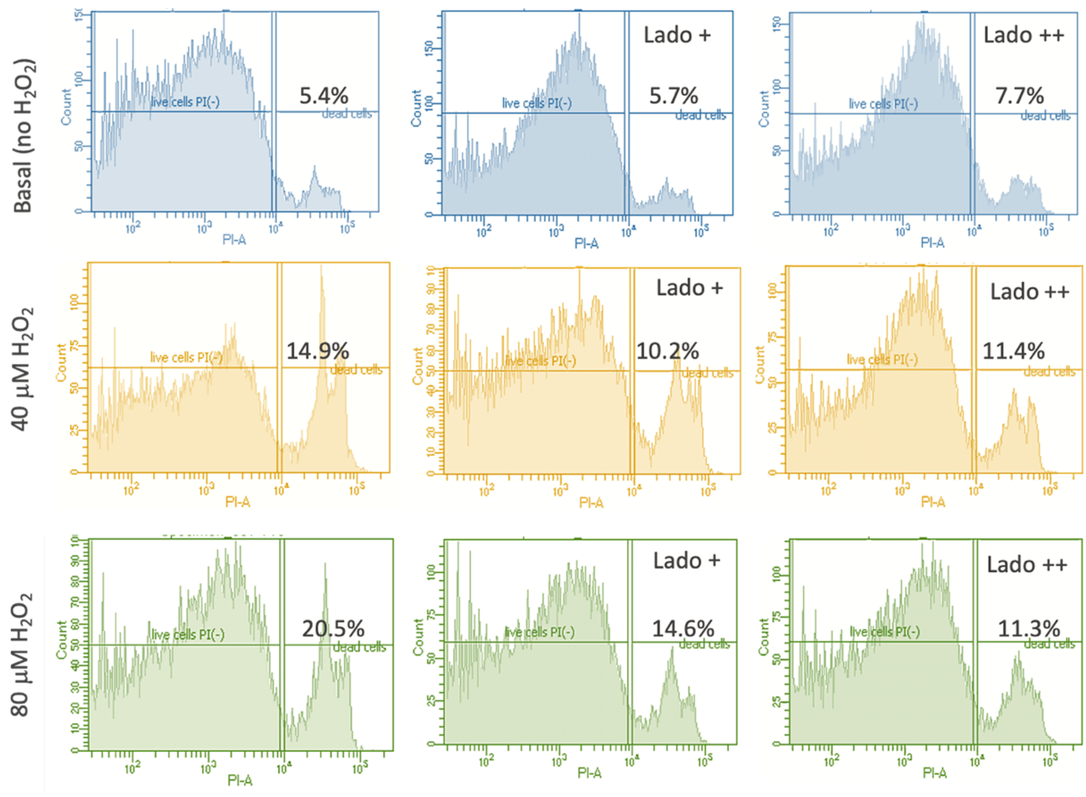

Figure S1. Flow cytometry analysis in naïve (untreated) and cells under oxidative stress condition in response to treatment with ladostigil (24 h). In each condition, 50 k cells were tested and split to live and death cell in basal condition (blue), following 40 M and 80 M H_2O_2 (yellow and green panels, respectively), and by the addition of ladostigil at 5.4 M (middle), 54 M (right). The data is of a representative experiment performed with 9 different biological samples using predetermined gates on the flow cytometry (shown by the vertical double lines in each panel. The high fluorescence of PI (x-axis, above the vertical line) indicates the fraction of dead cells (in % from total).
